# *EjFRI, FRIGIDA* (*FRI*) Ortholog from *Eriobotrya japonica*, Delays Flowering in Arabidopsis

**DOI:** 10.3390/ijms21031087

**Published:** 2020-02-06

**Authors:** Weiwei Chen, Peng Wang, Dan Wang, Min Shi, Yan Xia, Qiao He, Jiangbo Dang, Qigao Guo, Danlong Jing, Guolu Liang

**Affiliations:** 1 Key Laboratory of Horticulture Science for Southern Mountains Regions of Ministry of Education, College of Horticulture and Landscape Architecture, Southwest University, Beibei, Chongqing 400715, China; wenycww@163.com (W.C.);; 2 Academy of Agricultural Sciences of Southwest University, State Cultivation Base of Crop Stress Biology for Southern Mountainous Land of Southwest University, Beibei, Chongqing 400715, China

**Keywords:** *Eriobotrya japonica*, *FRIGIDA*, flowering time, expression pattern, ectopic expression

## Abstract

In the model species *Arabidopsis thaliana*, *FRIGIDA* (*FRI*) is a key regulator of flowering time and can inhibit flowering without vernalization. However, little information is available on the function in the Rosaceae family. Loquat (*Eriobotrya japonica*) belongs to the family Rosaceae and is a distinctive species, in which flowering can be induced without vernalization, followed by blooming in late-autumn or winter. To investigate the functional roles of *FRI* orthologs in this non-vernalization species, we isolated an *FRI* ortholog, dubbed as *EjFRI*, from loquat. Analyses of the phylogenetic tree and protein sequence alignment showed that EjFRI is assigned to eurosids I FRI lineage. Expression analysis revealed that the highest expression level of *EjFRI* was after flower initiation. Meanwhile, *EjFRI* was widely expressed in different tissues. Subcellular localization of EjFRI was only detected to be in the nucleus. Ectopic expression of *EjFRI* in wild-type Arabidopsis delayed flowering time. The expression levels of *EjFRI* in transgenic wild-type Arabidopsis were significantly higher than those of nontransgenic wild-type lines. However, the expression levels of *AtFRI* showed no significant difference between transgenic and nontransgenic wild-type lines. Furthermore, the upregulated *AtFLC* expression in the transgenic lines indicated that *EjFRI* functioned similarly to the *AtFRI* of the model plant Arabidopsis. Our study provides a foundation to further explore the characterization of *EjFRI*, and also contributes to illuminating the molecular mechanism about flowering in loquat.

## 1. Introduction

Flowering is a crucial phase during the life cycle of angiosperms. At present, the comprehensive flowering network is approached in the model plant species *Arabidopsis thaliana*, and extensive studies have been performed to describe related pathways of flower development [1]. In Arabidopsis, almost 180 genes have been illuminated in flowering-time control based on the analyses of transgenic plants and loss-of-function mutations [2]. To date, some regulatory genes of flowering time have been isolated and characterized from loquat, including *EjSOC1*, *EjFT*, *EjFD*, *EjGI*, *EjSVP*, *EjCO*, *EjAP1*, *EjTFL1* and *EjLEY* [3,4,5,6,7,8,9]. Among these genes, orthologous genes of the floral integrators, such as *EjFT* and *EjSOC1*, are identified and reported to regulate plant flowering-time by adjusting the environmental and endogenous pathways, including circadian clock regulation, photoreception, growth regulator of synthesis and response, and response to low temperatures [2,10,11].

Many flowering plants, which transfer from vegetative stage to reproductive growth, ensure themselves over winter vegetatively and flower in spring, after a long period of cold temperature [12,13,14]. The long-time cold treatment is called vernalization, which is also a major determinant of flowering time [15]. Plenty of information about the molecular mechanism of vernalization has been illuminated in Arabidopsis. Two dominant genes, *FRIGIDA* (*FRI*) and *FLOWERLING LOCUS C* (*FLC*), are required in the vernalization pathway [15,16,17]. Studies of natural variation and genetic analysis revealed that *FRI* encodes a nuclear protein which is found only in plants [13,18], and it plays a key role to delay flowering before vernalization, by upregulating the level of *FLC* mRNA [14,19,20]. The FRI protein is a novel nuclear protein including two coiled-coil regions, but has no conserved domains [20]. Although *FRI* serving as the determinant of flowering time has been deeply and extensively studied in the model plant, the functional characterization of *FRI* in horticulture plants, especially the fruit trees, needs further research. 

Loquat (*Eriobotrya* Lindl.) is a kind of tasty fruit with abundant nutrition and belongs to the Rosacea family [4,21]. Totally different from others Rosacea, the cultivated loquat (*Eriobotrya japonica* Lindl.) is a distinct species of genus *Eriobotrya* which need not undergo a chilling treatment and blooms in fall or early winter [22]. In this view, it is not known whether the function of the *FRIDIA* gene *in Eriobotrya japonica* is similar to the model plants. Hence, we sought to address this question by isolating *EjFRI* from floral buds of *Eriobotrya japonica.* Sequence and phylogenetic analyses revealed that EjFRI was assigned to the FRI orthologs in loquat. The expression pattern of *EjFRI* was investigated. The expression pattern analysis suggested that *EjFRI* did not have a similar trend to Arabidopsis during the development of loquat flowering. We also generated and analyzed Arabidopsis transgenic lines, which were expressed by 35S::*EjFRI*, to detect the function of *EjFRI* in flower development. Ectopic expression of *EjFRI* in Arabidopsis resulted in delayed flowering. Our study helps us better understand the role of *EjFRI* in the flower development of loquat and provides a foundation to explore the molecular mechanism about flowering in loquat.

## 2. Results

### 2.1. Isolation and Identification of FRI Orthologous Gene from Eriobotrya japonica

Based on the homology cloning and RACE techniques, 1386 bp full-length cDNA of *EjFRI* was isolated from *Eriobotrya japonica* (GenBank accession number: MN735437). It contains 1068 bp ORF, which encoded 355 amino acids (Supplementary Figure S1), as well as a 71 bp 5’- untranslated region (UTR) and 247 bp 3’-UTR. The molecular weight and isoelectric points of *EjFRI* are 38.3 kD and 8.24 (Supplementary Table S1), respectively.

**Figure 1:**
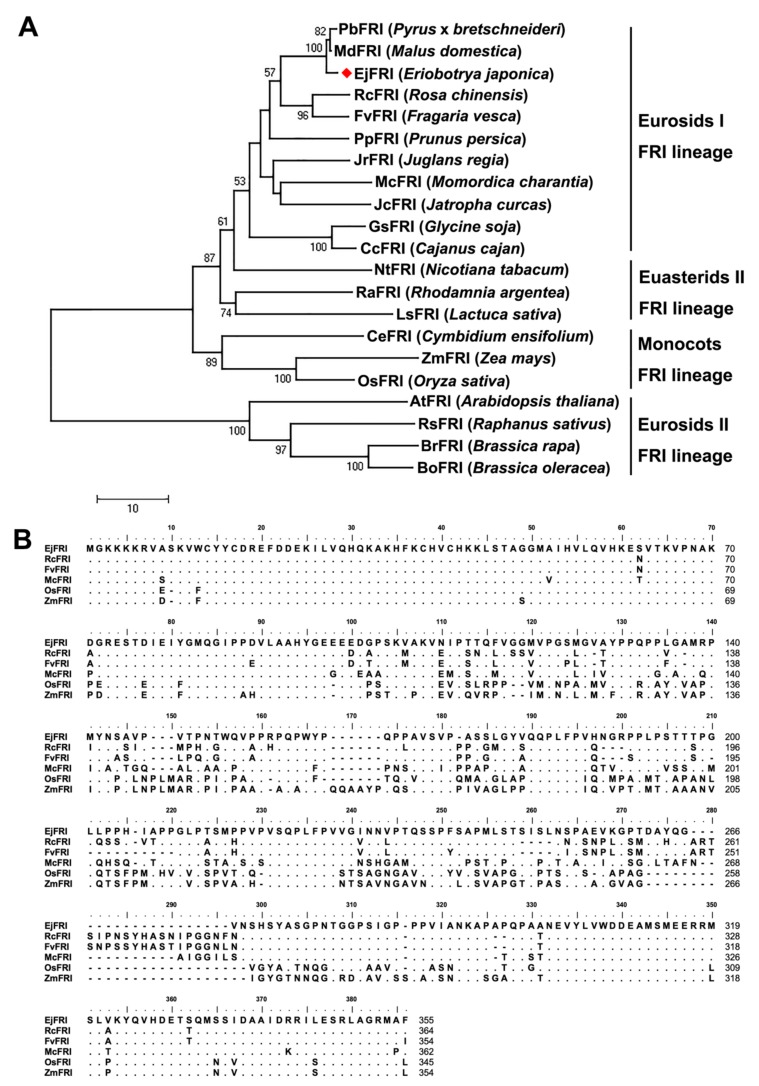
Sequence analysis of EjFRI. (**A**) Phylogenetic analysis of EjFRI protein orthologs. Sources of the orthologous proteins are indicated in Supplementary Table S2. The percentage of replicate trees in which the associated taxa clustered together in the bootstrap test (1000 replicates) is shown next to the branches. Bootstrap values >50 are shown on the tree. (**B**) Comparison of amino acid sequences of EjFRI with other reported FRIGIDA-like proteins in NCBI database. Amino acid residues identical to EjFRI are indicated as dots. To optimize the alignment, dashes were introduced into the sequence.

To reveal the evolution of the *EjFRI*, the cloned *EjFRI* sequence was then blasted with 21 FRI orthologs from other angiosperms (Supplementary Table S2). The phylogenetic analysis of EjFRI and other plants FRI amino acid sequences showed that EjFRI and the other FRIs from Rosaceae were grouped into eurosids I clade. Compared with PbFRI and MdFRI, they also belonged to a large clade with a high genetic relationship (Figure 1A). Amino sequence alignments revealed that EjFRI showed high similarity with its ortholog sequences (Figure 1B). These results suggest that EjFRI is FRI orthologs in loquat.

### 2.2. Spatiotemporal Expression of EjFRI in Loquat

**Figure 2:**
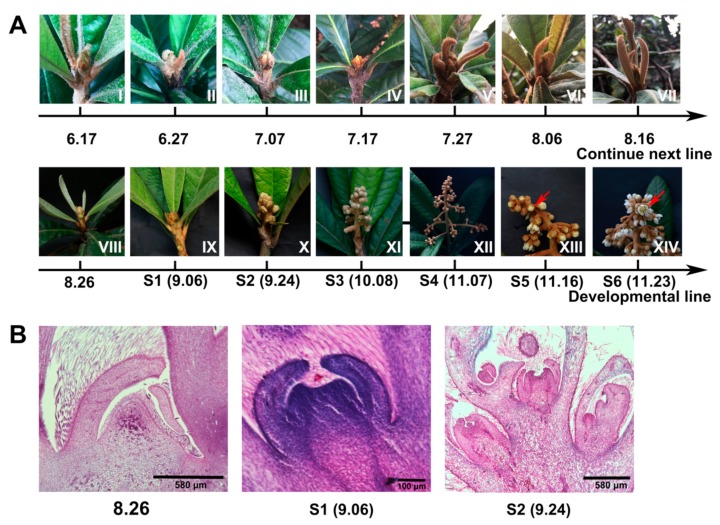
The morphology of tissues from June 17th to blooming. (**A**) June 17th to November 23th indicated the date for collecting apical buds. S1–S6 indicated the different stages of flowering development. S1: Floral meristems initiation. S2: Rapid panicle elongation. S3: Visible floral buds. S4: Branches of a panicle elongation. S5: White corollas of floral buds. S6: Full bloom. Red bars indicated the tissues of this stage. (**B**) Microscopic observations of apical buds for August 26th, S1 (9.06) and S2 (9.24).

**Figure 3:**
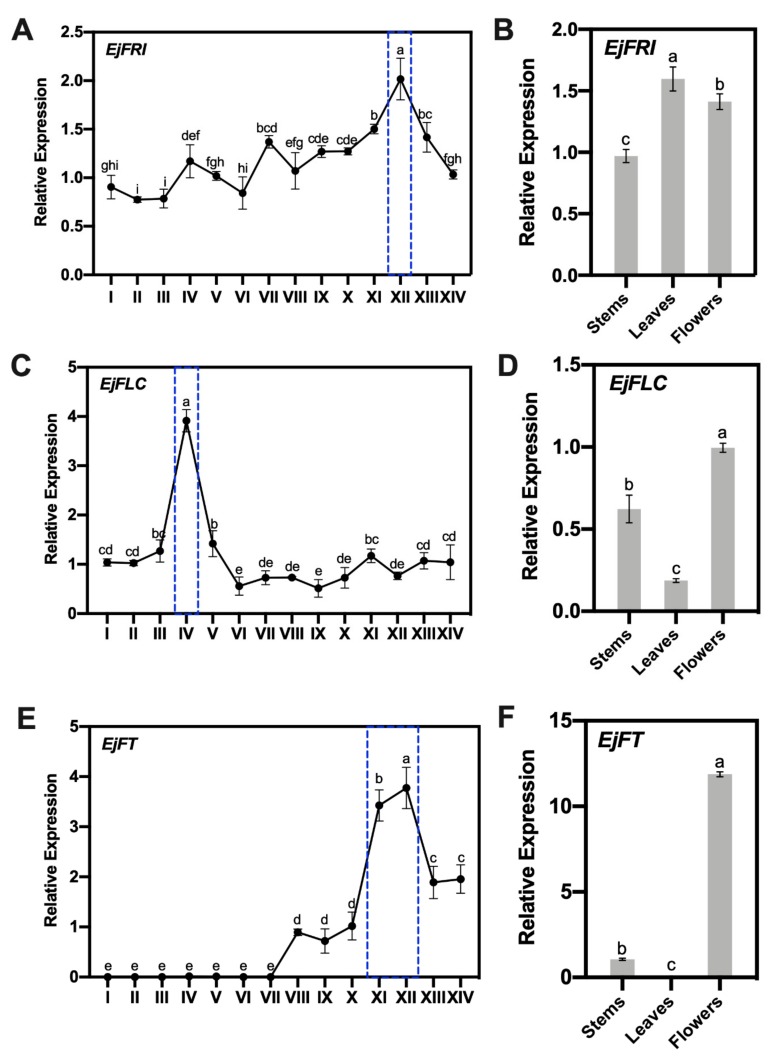
Relative expression patterns of *EjFRI* in loquat. (**A**,**C**,**E**) Relative expression levels of *EjFRI*, *EjFLC* and *EjFT* in the developmental stages of apical buds. Roman numerals (I–XIV) in the X-axis indicated the developmental stages of apical buds (see Figure 2). (**B**,**D**,**F**) Expression pattern of *EjFRI, EjFLC* and *EjFT* in different tissues of loquat. The stems, leaves and flowers used in B, D and F were collected on the same day, which was the full-bloom stage (November 23th). Data presented mean ± SD of three biological replicates. Letters above the bars indicate significant differences at *p* < 0.05 (Tukey–Kramer test).

Before vernalization, *FRIGIDA (FRI)* activates its target *FOWERING LOCUS C (FLC)* to delay flowering [14,23,24]. However, the cultivated loquat is harvested in summer and flowers in autumn and winter. Meanwhile, local expression of *FRI* in different tissues can also activate *FLC* to delay flowering [14], and *FRI* is a nonfunctional gene in summer annuals [24]. These possibilities encourage us to investigate the spatiotemporal expression of *EjFRI*, and thus we collected apical buds every 10 days after harvesting fruits, until flower initiation (Figure 2A, I–VIII). The tissues of different developmental flowers were also analyzed at the same time (Figure 2A, IX–XIV). The fourteen kinds of tissues included vegetative and reproductive developmental processes. Moreover, the analysis of paraffin-embedded sections of the apical buds (8.26, S1 and S2) revealed that the loquat began to transfer from the vegetative stage to reproductive growth at stage 1 (Figure 2B). In different periods of apical buds, the expression levels of *EjFRI* were fluctuant. However, in different developmental flowers, the expression level of *EjFRI* was stable from stage 1 to stage 2, and then it began to increase on stage 2 and reached the peak on stage 4, which was highlighted with a blue dotted box. Moreover, the expression level of stage 4 had a significant difference with the other tissues of different stages (Figure 3A). 

We also examined the expression pattern of *EjFRI* in specific tissues, including stems, leaves and flowers. *EjFRI* was expressed in all of these tissues, and it was the same as expression in Arabidopsis [14]. The highest and lowest expressions were observed in leaves and stems, respectively. In addition, there were significant differences among stems, leaves and flowers (Figure 3B). *FLC* is one of the two dominant genes in the vernalization pathway and could be directly activated by *FRI*, to delay flowering; it directly suppresses the expression of *FLOWERING LOCUS T* (*FT*), which is a floral pathway integrator [14,25]. Thus, the expression levels of *EjFLC* and *EjFT* were also analyzed. The result exhibited the highest level of *EjFLC* on July 17th, before flower initiation (Figure 3C). In specific tissues, the highest expression of *EjFLC* was detected in flowers. Meanwhile, the lowest expression of *EjFLC* was in leaves (Figure 3D), whereas the expression of *EjFT* was almost not expressed from June 17th to August 16th and in leaves (Figure 3E,F). The expression of *EjFT* began to increase on August 26th and showed high levels, which were maintained from stage 3 to stage 4 (Figure 3E). 

### 2.3. Subcellular Localization of EjFRI

**Figure 4:**
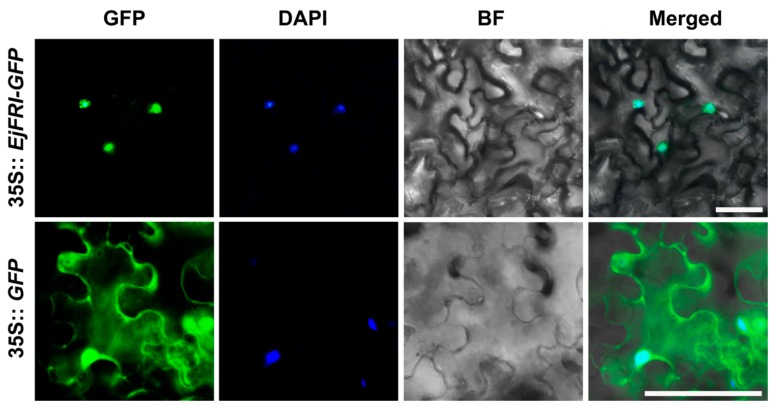
Subcellular localization of EjFRI. GFP, GFP fluorescence; 4,6-diamidino-2-phenylindole (DAPI) staining indicates nuclear localization; BF, bright-field; Merged, merged image of GFP and DAPI. Scale bars = 20 μm in 35S::*EjFRI-GFP* and 50 μm in 35S::*GFP*.

We generated 35S::*EjFRI-GFP* fusion protein to explore the subcellular localization of EjFRI. The 35S::*EjFRI-GFP* or 35S::*GFP* constructs harbored in *Agrobacterium tumefaciens cells* were transiently expressed in young leaves of tobacco (*Nicotiana benthamiana*), respectively. The fluorescence from 35S::*EjFRI-GFP* was detected only in the nucleus, but 35S::*GFP* control was localized both in the cytoplasm and nucleus (Figure 4). These results declared that EjFRI is a nuclear-localized protein, which is consistent with other previous results showing that FRI is localized in the nucleus [14,20,26].

### 2.4. EdFRI Delays Flowering in Arabidopsis

**Figure 5:**
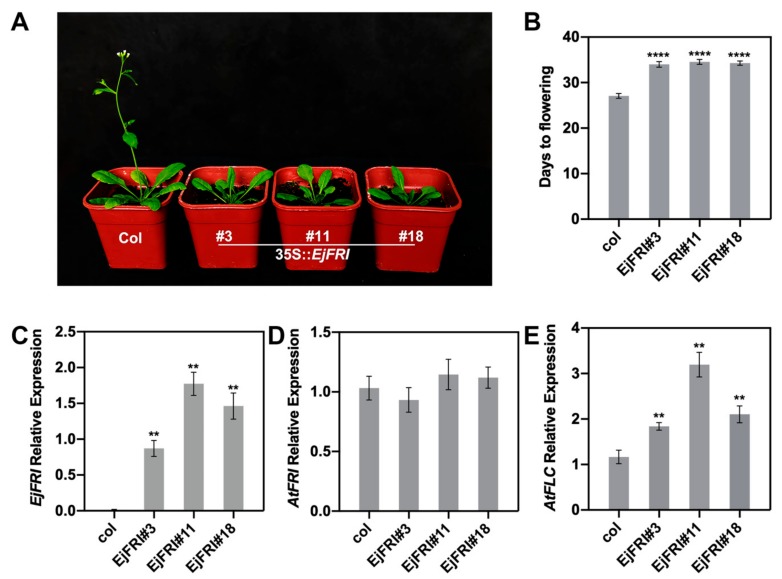
Overexpression of *EjFRI* delayed flowering Arabidopsis. (**A**) Transgenic plants delayed flowering compared to WT. (**B**) Flowering time grown under LD condition (Error bars indicating SD from 20 plants). (**C**,**D**,**E**) Expression levels of *EjFRI*, *AtFRI* and *AtFLC* in WT and transgenic plants (data represent mean ± SD of three biological replicates). The leaves of WT and transgenic plants were collected after flower bloom and used for qRT-PCR analysis. Asterisks indicate significant differences between WT and transgenic plants, ** *p* < 0.01, **** *p* < 0.0001, by Student’s *t*-test.

To explore the function of *EdFRI*, we generated 35S::*EjFRI* conduct and then transformed the conduct into *Arabidopsis* wild-type (WT) ecotype Col-0. Screening and detecting with kanamycin-selected and PCR identification (Supplementary Figure S2), we got a total of twenty-two 35S::*EjFRI* transgenic lines in WT *Arabidopsis* plants. Three T3 homozygous 35S::*EjFRI* transgenic lines were used to survey the flowering phenotype. Under long-day conditions, 35S::*EjFRI* T3 transgenic lines delayed flowering compared to WT (Figure 5A). This is the same with the result of *MsFRI-L* [27]. The three transgenic lines flowered at 34.37, 34.54 and 34 days after germination, respectively, while WT plants flowered at 27.09 days (Figure 5B). The expression levels of *EjFRI* in transgenic plants were higher than those of WT plants. However, there was no significant difference between transgenic and WT plants in regard to the expression levels of *Arabidopsis FRI* (*AtFRI*) (Figure 5C,D). We also examined the transcript levels of *AtFLC* in WT and transgenic plants. The expression levels of *AtFLC* were upregulated in transgenic plants (Figure 5E). These results indicated that *EjFRI* delayed flowering time in Arabidopsis and contributed to upregulating the *AtFLC.*

## 3. Discussion

Recent advances in genomics of plants have allowed us to isolate several of the flowering-control genes. Some of these genes are homologous to those which were previously characterized in Arabidopsis, and these researches have shown that their functions are conserved. Although some of them are orthologous genes of Arabidopsis, they have distinct functions or are not found in Arabidopsis [11]. In this study, EjFRI is the FRI orthologs shown as the phylogenetic analysis and amino sequences alignment (Figure 1A,B), but the structure of EjFRI protein is different from Arabidopsis. In Arabidopsis, the predicted protein of *FRI*, which was isolated from winter annuals accessions, has yet to show a specific protein or protein domain in the known databases [28]. It was only predicted to contain coiled-coil domains [11,13,29]. In *Brassica oleracea,* two *BoFRI* genes, *BolC.FRI.a and BolC.FRI.b*, were isolated. They are both orthologous to *AtFRI*, but one of them does not have the similar predicted structure of AtFRI [19]. BolC.FRI.a contains two coiled-coil domains, which is similar to AtFRI. However, there was only one coiled-coil domain of BolC.FRI.b. Interestingly, we didn’t find this similar structure in EjFRI. This situation also occurred in MdFRI (XM_008373696.3) and PbFRI (XM_009364983.1), which were grouped into a small clade with EjFRI in the phylogenetic tree (Figure 1A), as predicted by COILS (http://www.ch.embnet.org/software/COILS_form.html) [30]. It was reported that the loss of a predicted coiled-coil of BolC.FRI.b was induced by lower homology to the conserved region of AtFRI [19]. It also can explain why there were no coiled-coil structures in EjFRI, MdFRI and PbFRI. Because *Brassica* and *Arabidopsis* genera are in the same family (Brassicaceae) [31,32,33] and they have a close genetic relationship with each other, their FRI structures are similar. However, although *Eriobotrya*, *Malus* and *Pyrus* genera are in the same family (Rosaceae) [22,34,35], they have a distant genetic relationship with *Brassica* and *Arabidopsis* (Figure 1A). Hence, it was possible that there was no coiled-coil structure in EjFRI.

Studies on winter annuals plants have shown that their flowering time would be strongly delayed unless they were vernalized [36,37]. *FRI* (*FRIGIDA*) is a major factor that prevents plants from flowering rapidly without vernalization [13,36]. Unlike other Rosaceae plants [38,39,40,41,42,43], which lived through winters for flowering in springs, the flowering of cultivated loquat is not blocked, and they will bloom in late-autumn or winter. In woody perennials, shoot meristem displays transitions between vegetative and reproductive development [8]. We assumed that *EjFRI* may play a different role during this transition because of the exceptional flowering character of cultivated loquat. Therefore, the expression of *EjFRI* in different shoot meristems and different stages of flowering was carried out to validate this assumption. As we had assumed, the expression of *EjFRI* was fluctuated and did not have a single peak or a high expressing period during the transition from vegetative to reproductive development (Figure 2 and Figure 3A). It indicated that *EjFRI* did not have a distinct enrichment before flower bud differentiation. 

Interestingly, the expression of *EjFRI* reached a peak at stage 4, which was after the transition and before the full-bloom stage (Figure 2 and Figure 3A). Meanwhile, the loquat did not stop growth after stage 4; on the contrary, it continued to flower in one month, as usual. In Arabidopsis, *AtFRI* upregulates the expression of *AtFLC* and keeps it still at a high level, before vernalization, to delay flowering [10,11,44]. Although the highest expression of *EjFRI* was at stage 4, which was after flower bud differentiation and before the full-bloom stage, the loquat did not stop flowering in the cold winter as Arabidopsis. *EjFRI* probably plays roles in aspects of plant growth and development other than flowering [10]. Otherwise, *FRI* delays flowering by sustaining high expression levels of *FLC*, which encodes a MADS domain protein that suppresses flowering [23,45,46]. The expression of *FLC* would decrease in response to extended exposure to cold and remains low once *FLC* expression is reduced [44]. In our study, the expression of *EjFLC* enriched distinctly on July 17th and then remained low until the flower bloomed (Figure 3C). Although the low expression of *EjFLC* allowed the transition from vegetative to reproductive growth at the shoot apex (Figure 2A,B), it was not correlated with cold exposure, because the low expression of *EjFLC* began to decrease in the summer (Figure 3C). This phenomenon was also different from vernalization plants [12,39,47]. November 7th was the highest expression of *EjFRI*, and the expression of *EjFLC* was at a low level (Figure 3A,C), and this stage was after flower initiation (Figure 2A,B). It indicated that *EjFRI* was not directly upregulated for the expression of *EjFLC* to delay flowering, which was also different from the model plants [47,48,49]. Previous studies reported that FLC binds directly to the floral integrator gene *FT* to repress *FT* transcription [11,49]. In our study, *EjFT* was mainly detected in reproductive tissues, which was similar to the expression patterns of *EdFT* [4]. During the flowering development, the expression of *EjFT* increased from August 16th, when it was near to the date of flower initiation, and peaked on August 7th. It began to decrease when flowers were ready to fully bloom (Figure 2 and Figure 3E). The expression patterns of *EjFT* revealed that *EjFT* played a positive role in promoting flowering, which is similar to most of the identified *FT* homologs [50,51,52].

Previous studies have shown that *FRI* is widely distributed in different tissues which could perceive and transport the environmental signals, such as temperature and day length [10,14]. Moreover, in many plants, the environmental cues are important factors for the transition from vegetative to the reproductive stage [10]. In loquat, *EjFRI* was also expressed in different tissues of loquat, including stems, leaves and flowers. However, *EjFRI* exhibited some differences with the reported studies. Previously, in oilseed rape (*Brassica napus* L.), the expression of *BnaA.FRI.a* and *BnaA.FRI.d* ranged from barely detectable in stems and leaves to very high in flowers, and their expressions in flowers were markedly higher than in flower buds [53]. In Arabidopsis, the expression levels of *FRI* were similar in leaves and flowers [54]. On the contrary, in our study, *EjFRI* was detectable in stems and leaves, and there was a significant difference between leaves and flowers. These findings indicated that the expression patterns of *FRI* in different tissues may be related to the species.

Furthermore, *FRI* is a major determinant of flowering time in Arabidopsis, and it could directly activate *FLC* transcription to repress the expression of the floral activators, which are required to switch the shoot apical meristem from a vegetative to a reproductive fate, resulting in late flowering [45,47,48,49]. In our study, overexpression of *EjFRI* in Arabidopsis caused a late-flowering phenotype like the function of *AtFRI* (Figure 5A,B). This phenotype also occurred in transgenic Arabidopsis of *FRI* orthologous genes from *Brassica oleracea* and *Thellungiella halophila* when *BolC.FRI.a* and *ThFRI* were transformed into Columbia (Col-0), respectively [19,55,56]. We analyzed the relative expression of *EjFRI* and *AtFRI* at the same time to verify that the late-flowering phenotype was actually caused by *EjFRI* and not caused by *AtFRI* itself. The results indicated that the phenotype was genuinely induced by *EjFRI* (Figure 5C,D). In Arabidopsis, *FRI* delays flowering by upregulating its target gene, *FLC* [11,44,57]. Not surprisingly, *EjFRI* had also upregulated the expression of *AtFRI* (Figure 5E). We conclude that the data shown in our study could be deciphered through the functional conservation of *FRI* orthologs between *E. japonica* and other species [13,14,19,56].

## 4. Materials and Methods

### 4.1. Plant Materials

The tissues used were collected from mature loquat trees over 5 years old, named “Changbai No.1”, which were grown under natural conditions in the experimental farm of Southwest University, Chongqing, China (29°80′N, 106°40′E). The tissues were immediately frozen in liquid nitrogen after being collected. Col-0 (wild-type *A. thaliana* ecotype) was used for transgenic experiments. *Nicotiana benthamiana* was used for transient expression. *Arabidopsis* and *Nicotiana* were cultured under long-day conditions, which means 16 h of light and 8 h of dark, at 22 °C.

### 4.2. Gene Isolation and Sequence Analysis

Total RNA was obtained by using EASYspin Plant RNA Extraction kit (RN09, Aidlab, Beijing, China). RNA quality and concentration were detected with 1% RNase-free agarose gel electrophoresis and 2100 Bioanalyzer (Agilent Technologies, Santa Clara, CA, USA), respectively. First-strand cDNA was synthesized by using a PrimeScript^TM^ RT reagent Kit with gDNA Eraser (RR047A, TAKARA, Japan). According to the conserved regions of other reported *FRI* orthologs, such as *Malus* x *domestica* (XM_008373696.3) and *Pyrus* x *bretschneideri* (XM_009364983.1), we isolated partial sequences of *EjFRI* with the specific primers (FRIF and FRIR). The nested PCR was performed in both 5’- and 3’-rapid amplification of cDNA ends (RACE). Then, 2 μg of DNase I-treated RNA was serviced for 3’ RACE and 5’ RACE, using the 3’-Full RACE Core Set with PrimeScript^TM^ RTase (6101, TAKARA, Shiga, Japan) and SMARTer RACE 5’/3’ Kit Use Manual (Clontech, Mountain View, CA, USA). Finally, we designed the gene-specific primers (EjFRIF and EjFRIR) to amplify the full-length sequences named as *EjFRI*. All these PCR products were cloned into pMD19-T easy vector (TAKARA, Shiga, Japan) and then sequenced. The PCR primers mentioned above were listed in Supplementary Table S3.

Alignment of the deduced amino acid sequences was performed by using the ClustalW program. A phylogenic tree was constructed by using MEGA 5.0, under the Neighbor-Joining method, with 1000 bootstrap replicates. All the sequences used in the alignment and phylogenic tree are shown in Supplementary Table S2.

### 4.3. Vector Construction and Quantitative Real-Time PCR (qRT-PCR)

EjFRI coding regions were introduced into pBI121 (BD Biosciences Clontech, Mountain View, CA, USA), which is a binary vector, using *Xba*I and *Bam*HI restriction enzymes (TAKARA, Shiga, Japan). For the transient expression in *Nicotiana benthamiana*, 35S::*EjFRI*-GFP were constructed by cloning EjFRI into the modified pCAMBIA 1300-GFP vector, using *Bam*HI and *Sal*I restriction enzymes (TAKARA, Shiga, Japan) [58]. All the constructed vector were validated by sequencing and transformed into *Agrobacterium tumefaciens* strain GV3101-90 [59]. All the primers for vector constructions are shown in Supplementary Table S4.

Total RNA concentrations of different tissues were evaluated by using a Nano Drop 2000 Spectrophotometer (Thermo Scientific, Wilmington, DE, USA). First-strand cDNA was synthesized from 2 μg of DNase I-treated RNA, using a PrimeScript^TM^ RT reagent Kit with gDNA Eraser (RR047A, TAKARA, Shiga, Japan). Then, qRT-PCR was performed on qTOWER^3^ G (Analytik Jena, Jena, Germany), using the NovoStart® SYBR qPCR SuperMix Plus (E096-01A, novoprotein, Shanghai, China). The PCR system was followed: 95 °C for 30 s, followed by 40 cycles of 95 °C for 20 s and 56 °C for 1 min, and a melt cycle from 65 to 95 °C. After that, qEjFRI-F/R were used for detecting the spatiotemporal expression of *EjFRI*. As an internal control, the actin gene, qEjactin-F/R (JN004223), was used to normalized small differences in samples [60]. The gene expression level was analyzed by the 2^−∆∆Ct^ method described by Livak and Schmittegen [61]. The tissues of stage I–VIII were collected every 10 days after harvesting fruits until flower initiation. The tissues of stages IX–XIV (S1–S6), which represent different developmental flowers, were collected according to Jing et al. [62]. The stems, leaves and flowers used in B, D and F were collected on the same day, which was the full-bloom stage (November 23th). The primers of *EjFLC* for qRT-PCR were designed at the most conserved regions of the sequences of *MdFLC* (GenBank XM_008356596.2) and *PpFLC* (GenBank KP164015.1). The primers of *EjFT* for qRT-PCR referred to the previous report [4]. The primers used for spatiotemporal expression are shown in Supplementary Table S5.

### 4.4. Arabidopsis Transformation and Subcellular Localization Analysis

The 35S::*EjFRI* plasmids were transformed into *Arabidopsis* wild-type Col-0 by floral-dip method [63]. The seeds of transgene lines were selected on 1/2 MS medium with 50 μg/mL kanamycin. After 2 days of treatment at 4 °C, they were transferred to the long-day chambers (16 h light/8 h dark), at 22 °C, for 10 days. Then positive plants were transplanted in soil. T3 homozygous lines were used for observing phenotype and checking ectopic gene expression. The transgenic lines were detected by PCR and qRT-PCR analysis. The primers of 35S::*EjFRI* construct, which were TFRIF and TFRIR (Table S3), were used for PCR identification. The primers of qRT-PCR analysis were qRTEjFRI-F/R, qRTAtFRI-F/R and qRTAtFLC-F/R, respectively. The results were normalized against the expression of *TUB2* [25]. The qRT-PCR system was mentioned above. The leaves of WT and transgenic plants that were collected after flower bloom were used for qRT-PCR analysis. These primers used for qRT-PCR were shown in Supplementary Table S4.

To observe the subcellular localization of EjFRI, Agrobacterium-mediated transient of *N. benthamiana* leaves was used. The fluorescence signals of green fluorescent protein (GFP) were detected, using a fluorescence microscope Observer DP80 (Olympus, Tokyo, Japan). At the same time, a GFP-free construct was used as a negative control.

### 4.5. Data Analysis

Significant analysis in this study was executed by the Tukey–Kramer, Student *t*-test and SPSS software.

## 5. Conclusions

In this study, *EjFRI* was isolated, and its expression pattern and functional characterization of *EjFRI* were analyzed. As a special member of the Rosacea family, the *E. japonica* blooms without a long-time cold treatment. The expression levels of *EjFRI* did not have a significant enrichment before flower initiation, and the peak of the expression levels was during flower development. *EjFRI* was also not directly upregulated by the expression of *EjFLC*, which is its downstream gene that could regulate the transition from vegetative to reproductive development. These findings indicated that the expression pattern of *EjFRI* was different from the model plants and vernalization plants. We wonder if these differences may be caused by the distinct character of loquat or the far genetic relationship with the model plant. In addition, ectopic expression of *EjFRI* delayed flowering time and upregulated *AtFLC* in the transgenic lines. Our work improves our knowledge of *FRI* orthologous genes in *E. japonica* and provides a foundation for us to perform further investigations, to explore the molecular regulation mechanism of *EjFRI* in loquat.

## Supplementary Materials

Supplementary materials can be found at https://www.mdpi.com/1422-0067/21/3/1087/s1.
