# Supplementary material for: EjFRI, FRIGIDA (FRI) Ortholog from Eriobotrya japonica, Delays Flowering in Arabidopsis
Source: Int J Mol Sci. 2020 Feb 6;21(3):1087. doi: 10.3390/ijms21031087 (PMC7038142; doi:10.3390/ijms21031087)
Supplement: Supplementary file 1 [file ijms-21-01087-s001.pdf]

International Journal of *Molecular Sciences*

MDPI

Supplementary

Table S1. The molecular weight and isoelectric points of EjFRI protein

Protein	Molecular weight	Theoretical isoelectric points
EjFRI	38.3 kD	8.24

Table S2. Names and accession numbers used for phylogenetic analysis

Name	Species	Accession number
PbFRI	*Pyrus* x *bretschneideri*	XM_009364983.1
MdFRI	*Malus domestica*	XM_008373696.3
EjFRI	*Eriobotrya japonica*	MN735437
RcFRI	*Rosa chinensis*	XM_024343890.1
FvFRI	*Fragaria vesca*	XM_004293767.2
PpFRI	*Prunus persica*	XM_020562035.1
JrFRI	*Juglans regia*	XM_018957193.1
McFRI	*Momordica charantia*	XM_022290834.1
JcFRI	*Jatropha curcas*	XM_012233176.2
GsFRI	*Glycine soja*	XM_028378209.1
CcFRI	*Cajanus cajan*	XM_020365670.2
NtFRI	*Nicotiana tabacum*	XM_016645985.1
RaFRI	*Rhodamnia argentea*	XM_030659311.1
LsFRI	*Lactuca sativa*	XM_023889701.1
CeFRI	*Cymbidium ensifolium*	MK470627.1
ZmFRI	*Zea mays*	NM_001143503.1
OsFRI	*Oryza sativa*	XM_015756342.2
AtFRI	*Arabidopsis thaliana*	AF228500
RsFRI	*Raphanus sativus*	KY069028.1
BrFRI	*Brassica rapa*	HQ615935.1
BoFRI	*Brassica oleracea*	JN191450.1

Table S3. Primers used for gene cloning

Primer name	Primer sequences (5′-3′)	Purpose of primers	Annealing temperatures℃
FRIF	AGAGCCGCAGAAGGAGGGCA	Partial sequences Forward Primer	58
FRIR	GGAATACTTGTTGAAAGCAT	Partial sequences Reverse Primer	
GSPFRI-1	GTGCACAATGGGAGACCACCTCTA	3′RACE Gene Specific Outer Primer	56
3′RACE Outer	TACCGTCGTTCCACTAGTGATTT	3′RACE Adaptor Outer Primer	

GSPFRI-2	GCTCCCATGCTTTCAACAAGTATTC	3′RACE Gene Specific Inner Primer	56
3′RACE Inner	CGCGGATCCTCCACTAGTGATTTCACTATAGG	3′RACE Adaptor Inner Primer	
FRIGSP1	TGGAGGCTGTGGATACCATGGC	5′RACE Gene Specific Outer Primer	56
5′RACE Outer	TAATACGACTCACTATAGGGCAAGCAGTGGTATCAACGCAGAGT	5′RACE Adaptor Outer Primer	
FRIGSP2	CTCTTCCTCTCCATAATGTGC	5′RACE Gene Ppecific Inner Primer	56
5′RACE Inner	CTAATACGACTCACTATAGGGC	5′RACE Adaptor Inner Primer	
EjFRIF	ATGGGAAAGAAGAAGAAGAGAGT	Full-length Forward Primer	60
EjFRIR	CTAAAATGCCATTCGACCAGCAAGC	Full-length Reverse Primer	

Table S4. Primers used for vector construction

Primer name	Primer sequences (5′-3′)	Purpose of primers	Annealing temperatures℃
TFRIF	GCTCTAGAATGGGAAAGAAGAAGAAGAGAGTGG<br>*XBaI*	pBI121 vector	56
TFRIR	CGCGGATCCCTAAAATGCCATTCGACCAGCAAG<br>*BamHI*		
LFRIF	GGATCCATGGGAAAGAAGAAGAAGAGAGT<br>*BamHI*	pCAMBIA1300 -GFP vector	60
LFRIR	GTCGACAAATGCCATTCGACCAGCAAGC<br>*SalI*		

Table S5. Primers used for quantitative real-time PCR (qRT-PCR)

Primer name	Primer sequences (5′-3′)	Annealing temperatures℃
qEjFRI-F	GCAGCACCAGAAAGCCAAGCA	56
qEjFRI-R	GTTGACTCCCTGCCATCCTTCG	
qEjFLC-F	GAAATGCATACCCCGATCCCT	56
qEjFLC-R	TCTCATATCCTGCGGTTGCT	
qEjFT-F	GTTGTTGGACGAGTGGTAG	56
qEjFT-R	TAACCTCTTTATTGCCGTAG	
qEjactin-F	AATGGAACTGGAATGGTCAAGGC	56
qEjactin-R	TGCCAGATCTTCTCCATGTCATCCCA	
qRTEjFRI-F	GCGTCGAAGGTGTGGTGTTAC	56
qRTEjFRI-R	ATGGCCATGCCGCCAGCGGTG	

qRTAtFRI-F	GTTAGGAAGAAGTGTATATGC	56
qRTAtFRI-R	GTACTGTAGTGGATGTCTCTGTC	
qRTAtFLC-F	GACTGCCCTCTCCGTGACTAG	56
qRTAtFLC-R	ATGATGATTATTCTCCATCTGGC	
TUB2-F	ATCCGTGAAGAGTACCCAGAT	56
TUB2-R	AAGAACCATGCACTCATCAGC	

```
1      ATGGGAAAGAAGAAGAAGAGAGTGGCGTCGAAGGTGTGGTGTTACTACTGCGATAGAGAATTCGACGATGAGAAGATA
1       M  G  K  K  K  K  R  V  A  S  K  V  W  C  Y  Y  C  D  R  E  F  D  D  E  K  I

79     CTGGTGCAGCACCAGAAAGCCAAGCACTTCAAGTGCCATGTCTGCCATAAAAAGCTCTCCACCGCTGGCGGCATGGCC
27      L  V  Q  H  Q  K  A  K  H  F  K  C  H  V  C  H  K  K  L  S  T  A  G  G  M  A

157    ATTCACGTCCTCCAGGTCCACAAAGAGAGCGTCACCAAGGTTCCCAATGCGAAGGATGGCAGGGAGTCAACGGATATT
53      I  H  V  L  Q  V  H  K  E  S  V  T  K  V  P  N  A  K  D  G  R  E  S  T  D  I

235    GAAATTTATGGGATGCAAGGAATCCCACCTGACGTCTTGGCTGCACATTATGGAGAGGAAGAGGAAGATGGTCCATCA
79      E  I  Y  G  M  Q  G  I  P  P  D  V  L  A  A  H  Y  G  E  E  E  E  D  G  P  S

313    AAAGTAGCTAAAGTAAACATCCCAACAACCCAGTTTGTTGGTGGTATGGTGCCAGGTTCGATGGGGGTTGCATATCCT
105     K  V  A  K  V  N  I  P  T  T  Q  F  V  G  G  M  V  P  G  S  M  G  V  A  Y  P

391    CCCCAACCACCTTTAGGTGCAATGCGGCCAATGTACAATTCTGCAGTTCCAGTGACTCCGAATACTTGGCAAGTTCCA
131     P  Q  P  P  L  G  A  M  R  P  M  Y  N  S  A  V  P  V  T  P  N  T  W  Q  V  P

469    CCTCGTCCCCAGCCATGGTATCCACAGCCTCCAGCAGTCTCAGTACCTGCTTCCTCATTGGGTTATGTGCAGCAGCCA
157     P  R  P  Q  P  W  Y  P  Q  P  P  A  V  S  V  P  A  S  S  L  G  Y  V  Q  Q  P

547    TTGTTTCCTGTGCACAATGGGAGACCACCTCTACCATCAACCACCACCCCTGGACTTCTGCCTCCGCATATAGCCCCT
183     L  F  P  V  H  N  G  R  P  P  L  P  S  T  T  T  P  G  L  L  P  P  H  I  A  P

625    CCTGGCCTTCCTACATCCATGCCTCCTGTTCCTGTATCACAACCCCTATTTCCTGTTGTTGGTATAAATAATGTACCA
209     P  G  L  P  T  S  M  P  P  V  P  V  S  Q  P  L  F  P  V  V  G  I  N  N  V  P

703    ACTCAAAGTTCTCCCTTTTCCGCTCCCATGCTTTCAACAAGTATTTCCCTAAATTCTCCAGCTGAAGTTAAGGGACCA
235     T  Q  S  S  P  F  S  A  P  M  L  S  T  S  I  S  L  N  S  P  A  E  V  K  G  P

781    ACGGATGCTTATCAAGGTGTTAATTCACACTCTTATGCATCTGGTCCAAACACCGGTGGTCCATCAATTGGACCACCC
261     T  D  A  Y  Q  G  V  N  S  H  S  Y  A  S  G  P  N  T  G  G  P  S  I  G  P  P

859    CCTGTTATTGCAAACAAAGCTCCTGCTCCCCAGCCAGCTGCTAATGAGGTGTATCTAGTTTGGGATGATGAAGCTATG
287     P  V  I  A  N  K  A  P  A  P  Q  P  A  A  N  E  V  Y  L  V  W  D  D  E  A  M

937    TCCATGGAGGAAAGAAGAATGTCCTTAGTGAAGTATCAGGTTCATGATGAAACTAGCCAGATGAGTTCAATCGATGCA
313     S  M  E  E  R  R  M  S  L  V  K  Y  Q  V  H  D  E  T  S  Q  M  S  S  I  D  A

1015   GCCATAGACAGAAGGATTTTGGAGAGCAGGCTTGCTGGTCGAATGGCATTTTAG
339     A  I  D  R  R  I  L  E  S  R  L  A  G  R  M  A  F  *
```

Figure S1. Nucleotide and amino acid sequence of *EjFRI*

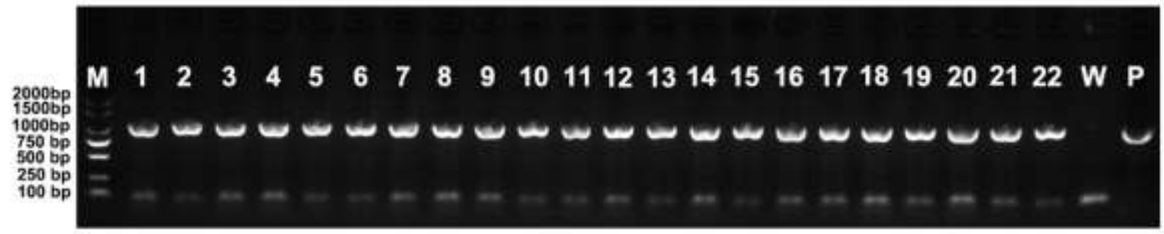

Figure S2. PCR identification of positive transgenic lines. M: Maker; Lane 1-22: *EjFRI* transgenic plants; W: Wild-type *Arabidopsis*; P: Transgenic plasmid.

© 2019 by the authors. Submitted for possible open access publication under the terms and conditions of the Creative Commons Attribution (CC BY) license (http://creativecommons.org/licenses/by/4.0/).
